# Alcohol-Use Disorder and Severe Mental Illness

**Published:** 1996

**Authors:** Robert E. Drake, Kim T. Mueser

**Affiliations:** Robert E. Drake, M.D., Ph.D., is a professor in both the Department of Psychiatry and the Department of Community and Family Medicine at Dartmouth Medical School and director of the New Hampshire-Dartmouth Psychiatric Research Center, Concord, New Hampshire. Kim T. Mueser, Ph.D., is an associate professor in both the Department of Psychiatry and the Department of Community and Family Medicine at Dartmouth Medical School and a senior researcher at the New Hampshire-Dartmouth Psychiatric Research Center, Concord, New Hampshire

**Keywords:** AODD (alcohol and other drug use disorder), behavioral and mental disorder, schizophrenia, affective psychosis, comorbidity, prevalence, etiology, sociocultural AODC (causes of AOD use, abuse, and dependence), disease course, patient assessment, treatment, psychiatric care, prevention of AODR (alcohol and other drug related) problems

## Abstract

Alcohol-use disorders (AUD’s) commonly occur in people with other severe mental illnesses, such as schizophrenia or bipolar disorder, and can exacerbate their psychiatric, medical, and family problems. Therefore, to improve detection of alcohol-related problems, establish correct AUD diagnoses, and develop appropriate treatment plans, it is important to thoroughly assess severely mentally ill patients for alcohol and other drug abuse. Several recent studies have indicated that integrated treatment approaches that combine AUD and mental health interventions in comprehensive, long-term, and stagewise programs may be most effective for these clients.

Alcohol-use disorder^1^ (AUD) is the most common co-occurring disorder in people with severe mental illnesses, such as schizophrenia and bipolar disorder. This article reviews several aspects of AUD among mentally ill patients—prevalence and etiology, clinical correlates, course and outcome, assessment, and treatment—emphasizing practical clinical implications within each of these categories. Because people with AUD also frequently suffer from other drug-use disorders with similar clinical correlates, similar impacts on the course of mental illnesses, and similar principles of treatment as AUD, the information summa- rized in this article pertains to the broader problem area of alcohol and other drug (AOD)-use disorders among people with severe psychiatric disorders.

## Prevalence and Etiology

Severe mental disorders frequently are complicated by comorbid disorders, such as medical illnesses, mental retardation, and AOD abuse. Co-occurring AOD-use disorders represent the most frequent and clinically most significant comorbidity among mentally ill patients, and alcohol is the most commonly abused drug (Cuffel 1996). Undoubtedly, the fact that alcohol is readily available and that its purchase and consumption are legal for anyone age 21 and older contributes to its widespread abuse. For example, in community samples evaluated for the Epidemiologic Catchment Area (ECA) study, 33.7 percent of people diagnosed with schizophrenia or schizo-phreniform disorder and 42.6 percent of people with bipolar disorder also met the lifetime criteria for an AUD diagnosis, compared with 16.7 percent of people in the general population (Regier et al. 1990). Furthermore, according to the National Comorbidity Study, people with mania are 9.7 times as likely as the general population to meet the lifetime criteria for alcohol dependence (Kessler et al. 1996).

Because of the ways in which AOD-use disorders complicate severe mental illness, comorbidity rates tend to be particularly high among young males and clients in high-risk settings, such as hospitals, emergency rooms, and homeless shelters. The high rates of AOD-use disorders, especially among young adults, may be due partly to changes in the United States’ mental health care system during the past few decades. An entire generation of people with severe mental illnesses developed their disorders during the era of deinstitutionalization. These people resided predominantly in their communities rather than in hospitals; they received few vocational, recreational, and social opportunities but experienced regular exposure and ready access to AOD’s. As a result, the rates of diagnosed AOD-use disorders in mental health settings have continued to rise. In addition, clinicians have become more aware of the high prevalence of AOD-use disorders and more skilled at identifying them (Cuffel 1996).

Although people with severe mental illnesses probably experiment with AOD’s for the same reasons as other members of the general population, several additional factors may contribute to the elevated rates of AOD-use disorders among severely mentally ill people. These factors include a downward social drift into poor, urban living settings, resulting in increased exposure and access to AOD’s; attempts to alleviate, or self-medicate, the symptoms of mental illness, the side effects of psychotropic medications, and the dysphoria associated with mental illness; and attempts to avoid being labeled a “mental patient” (Minkoff and Drake 1991). Other factors involved in the underlying mechanisms (i.e., the etiology) of AOD-use disorders for this population may include early experimentation due to social pressure; desire to experience alcohol’s short-term effects, such as relief of anxiety; and clinical correlates, such as antisocial behavior.

Although more research must be conducted on the etiology of AOD-use disorders in mentally ill people, most likely these disorders are determined, as in other people, by a complex set of biological, psychological, and social (i.e., biopsychosocial) factors. However, distinguishing the causes of AOD-use disorders from factors that sustain AOD use or that are correlates or consequences of AOD use often is difficult. For example, the following items appear to be related to sustained AUD, regardless of the reasons for initial alcohol use: positive reinforcement in the brain’s reward system; association with internal or external cues through classical conditioning; poor cognitive, social, and vocational functioning; and lack of significant social and material resources (Donovan 1988).

## Social and Psychological Correlates of AUD

Several studies have indicated that AUD among people with severe mental illnesses is associated with various manifestations of poor psychological and social adjustment (Dixon et al. 1990; Drake et al. 1989; Kozaric-Kovacic et al. 1995). These manifestations include relapses of psychiatric symptoms; psychosocial instability; other drug-use disorders; disruptive behavior; medical problems, such as HIV infection; family problems (e.g., in managing finances or maintaining positive relationships with family members); and institutionalization in hospitals and jails. Moreover, patients with dual diagnoses of severe mental illness and AUD are particularly prone to unstable housing arrangements and homelessness (see sidebar, pp. 90–91). Finally, dually diagnosed patients tend to be noncompliant with outpatient treatment and frequently receive health services in emergency rooms, hospitals, and jails (Bartels et al. 1993). Not all these correlates, however, have been observed consistently (e.g., the exacerbation of schizophrenic symptoms), and some correlates (e.g., violence or HIV infection) may be linked more closely with the abuse of drugs other than alcohol.

**Homelessness and Dual Diagnosis**

Homeless people with co-occurring severe mental illnesses and alcohol-use disorder (AUD) represent a particularly vulnerable subgroup of the homeless with complex service needs (Drake et al. 1991). Although often referred to as dually diagnosed, these people typically are impaired by several additional problems, including abuse of drugs other than alcohol, general medical illnesses, and legal problems. This group also has histories of trauma and behavioral disorders, deficient social and vocational skills, and support networks that include people involved in alcohol and other drug (AOD) abuse or other illegal behavior. Compared with other homeless subgroups, those with co-occurring severe mental illnesses and AUD are more likely to experience harsh living conditions, such as living on the streets rather than in shelters; suffer from psychological distress and demoralization; grant sexual favors for food and money; be picked up by police; become incarcerated; be isolated from their families; and be victimized (Fischer 1990).

Much of our current knowledge of homeless adults with dual disorders comes from National Institute on Alcohol Abuse and Alcoholism initiatives funded by the Stewart B. McKinney Act (Huebner et al. 1993). These initiatives include a 3-year, 14-project demonstration to develop, implement, and evaluate interventions for homeless adults with AOD-related problems. Two of the projects specifically have targeted homeless people with co-occurring severe mental illnesses and AOD-use disorders.

***Prevalence and Etiology***

In a comprehensive review, Fischer (1990) found that between 3.6 and 26 percent of homeless adults suffered from both a mental disorder and AUD. The rates of co-occurring mental and AOD-use disorders ranged from 8 to 31 percent. Other recent reviews also have determined that the rates of dual diagnoses among the homeless range from 10 to 20 percent (Drake et al. 1991).

Many studies investigating the causes (i.e., etiology) of homelessness and dual diagnoses have suggested that people with co-occurring mental and AOD-use disorders are particularly prone to losing family supports and stable housing and becoming homeless (Drake et al. 1991). One reason for this increased risk appears to be that dually diagnosed clients often are excluded from housing and treatment programs designated specifically for people with single disorders (Drake et al. 1991).

***Management of Homeless People With Dual Diagnoses***

Several consistent themes have emerged in the literature on interventions for homeless people with dual disorders. Most important, interventions should focus primarily on meeting the clients’ basic needs related to subsistence and safety. Moreover, appropriate interventions should provide needed structure, support, and protection. Specific treatment recommendations include the following (Drake et al. 1991):

- Integration of mental health and substance abuse interventions—for example, through intensive case management and group interventions
- Provision of services to families as well as to individual clients
- Development of culturally relevant services
- Development of long-term, stagewise interventions.

Recent studies have examined the integration of mental health, AOD abuse, and housing interventions in various configurations. These studies show that both engaging and retaining dually diagnosed homeless people in treatment programs are extremely difficult, especially in short-term or residential programs (Blankertz and Cnaan 1994; Burnam et al. 1995; Rahav et al. 1995). Furthermore, any gains that the clients make during short-term or residential treatment tend to erode rapidly following discharge. Several observations may help explain these findings. For example, behaviors that may represent common adaptations to homeless living, such as intimidating or threatening other people, often are incompatible with participation in treatment and recovery programs (Weinberg and Koegel 1995). Homeless people also often have difficulty participating in treatment or rehabilitation before they have attained some measure of stable subsistence (Baxter and Hopper 1981). Finally, rehabilitation and recovery are long-term endeavors that take years for most dually diagnosed people. Consequently, programs that first address the clients’ subsistence needs and then provide long-term treatment in progressive stages are best suited for dually diagnosed homeless people (Drake et al. 1994).

***Summary***

Among the homeless, those with severe mental illnesses and co-occurring AUD constitute a complex subgroup. Meeting their needs requires an intensive effort over months or years, with multidisciplinary teams providing outreach; addressing subsistence needs; integrating mental health, substance abuse, and housing interventions; and allowing for a longitudinal, stagewise recovery process. Because researchers have identified some of the pathways by which dually diagnosed individuals frequently become homeless, interventions to prevent homelessness also may be possible. Such preventive interventions could focus on unstable housing situations and evictions, more careful discharge planning from institutional settings, greater support for families, more efficient use of resources, and help with money management (Substance Abuse and Mental Health Services Administration 1996).

—*Robert E. Drake and Kim T. Mueser*

ReferencesBaxterEHopperKPrivate Lives/Public Spaces: Homeless Adults on the Streets of New York CityNew YorkCommunity Service Society1981BlankertzLECnaanRAAssessing the impact of two residential programs for dually diagnosed homeless personsSocial Service Review685365601994BurnamMAMortonSCMcGlynnEAPetersenLPStecherBMHayesCVaccaroJVAn experimental evaluation of residential and nonresidential treatment for dually diagnosed homeless adultsJournal of Addictive Diseases1441111341995892993610.1300/j069v14n04_07DrakeREOsherFCWallachMAHomelessness and dual diagnosisAmerican Psychologist46114911581991177215210.1037//0003-066x.46.11.1149DrakeREYovetichNABeboutRRHarrisMMcHugoGJIntegrated Treatment for Dually Diagnosed Homeless AdultsPaper presented at the NIAAA Cooperative Agreement Program for Homeless Persons with Alcohol and Other Drug Problems meetingRockville, MDSeptember 1994FischerPJAlcohol and Drug Abuse and Mental Health Problems Among Homeless Persons: A Review of the Literature, 1980–1990Rockville, MDNational Institute on Alcohol Abuse and Alcoholism and National Institute of Mental Health1990HuebnerRBPerlHIMurrayPMScottJETutunjianBAThe NIAAA Cooperative Agreement Program for Homeless Persons with Alcohol and Other Drug Problems: An overviewAlcoholism Treatment Quarterly103/45201993RahavMRiveraJJNuttbrockLNg-MakDSturzELLinkBGStrueningELPepperBGrossBCharacteristics and treatment of homeless, mentally ill, chemical-abusing menJournal of Psychoactive Drugs27931031995760244510.1080/02791072.1995.10471677Substance Abuse and Mental Health Services AdministrationCooperative Agreements for DMHS/ CSAT Collaboration Program to Prevent HomelessnessGFA No. SM 96–01Rockville, MDU.S. Department of Health and Human Services1996WeinbergDKoegelPImpediments to recovery in treatment programs for dually diagnosed homeless adults: An ethnographic analysisContemporary Drug Problems221932361995

Although one is tempted to regard AUD as the cause of the above-mentioned social and psychological problems, many additional factors may contribute to poor adjustment. For example, alcohol-abusing patients with mental disorders also are prone to abuse other potentially more toxic drugs, to be noncompliant with medications, and to live in stressful circumstances without strong support networks (Drake et al. 1989). Moreover, these patients may differ premorbidly from patients with the same mental disorders who do not abuse drugs. Laboratory experiments may help clarify some of the relationships between AUD and poor adjustment, but the circumstances, quality, and quantity of alcohol use in a laboratory may differ significantly from the typical alcohol-use patterns of people in the community (Dixon et al. 1990). Support for the role of AUD in causing poor adjustment, however, comes from findings indicating that severely mentally ill patients who become abstinent show many signs of improved well-being. These patients either resemble severely mentally ill people who have never experienced AUD (Drake et al. 1996*a*) or rate between non-AOD users and current users on many clinical and functional measures (Kovasznay 1991; Ries et al. 1994).

## Course and Outcome

Data regarding the course and outcome of co-occurring mental illness and AUD are accumulating rapidly. Short-term studies (i.e., those lasting 1 year or less) of patients in traditional treatment systems indicate that these dually diagnosed people are prone to negative outcomes, such as continuing AUD, as well as to high rates of homelessness, disruptive behavior, psychiatric hospitalization, and incarceration. For example, outpatients with schizophrenia and co-occurring AUD had twice the rate of hospitalization during 1-year followup compared with patients with only schizophrenia (Drake et al. 1989). Fewer studies have been conducted on the long-term outcomes (i.e., results more than 1 year later), but findings tend to show persistent AUD and poor adjustment (Drake et al. 1996*a*; Kozaric-Kovacic et al. 1995).

Conversely, dually diagnosed patients who achieve abstinence appear to experience better prognoses and more positive adjustment, including improved psychiatric symptoms and decreased rates of hospitalization. For example, ECA study participants with schizophrenia and AUD who attained abstinence had decreased rates of depression and hospitalization at 1-year followup (Cuffel 1996). These optimistic findings have fueled attempts to develop more effective AUD interventions among psychiatric patients (see the section “Treatment”).

## Assessment

Thorough AOD-use assessment includes three overlapping but conceptually separable tasks: detection, diagnosis, and treatment planning (Drake et al. 1996*b*). Detection refers to the identification of harmful or dangerous alcohol-use patterns, whether or not they fulfill the criteria of abuse or dependence. Conversely, diagnosis denotes the assignment of a label of AOD-use disorder, based on the criteria of the American Psychiatric Association’s *Diagnostic and Statistical Manual of Mental Disorders* (DSM). Treatment planning entails a more thorough analysis of the biopsychosocial factors sustaining AOD abuse and a specific plan to address them.

### Detection

Numerous studies have shown that AOD-use disorders typically are underdiagnosed in acute-care psychiatric settings (Drake et al. 1993*a*). Several factors account for the high rates of nondetection, including mental health clinicians’ inattention to AOD abuse; patients’ denial, minimization, or inability to perceive the relationships between AOD use and their medical and social problems; and the lack of reliable and valid detection methods for this population. Failure to detect AOD abuse in psychiatric settings can result in mis-diagnosis; overtreatment of psychiatric syndromes with medications; neglect of appropriate interventions, such as detoxification, AOD education, and AOD abuse counseling; and inappropriate treatment planning.

Several procedures could improve the detection of AOD-use disorders and of potentially harmful AOD use among psychiatric patients. For example, mental health clinicians should be educated about AOD’s and, subsequently, should maintain both a high index of suspicion for AOD-use disorders and an awareness of their clinical correlates. Little evidence exists indicating that psychiatric patients can sustain moderate AOD use over long periods of time without incurring problems (Drake et al. 1996*a*), although AOD use without abuse may occur at any time (Lehman et al. 1996). Consequently, clinicians should pay attention to any current AOD use, even if there appear to be no harmful consequences. Furthermore, clinicians should pay attention to reports of clients’ past AOD-related problems, because the clients are more likely to report past use than current use (Barry et al. 1995).

Multiple tools are available that detect the majority of mentally ill people who abuse alcohol. These tools include brief screening tests, such as the CAGE and the Michigan Alcoholism Screening Test (MAST). Other standard detection approaches include assessment using more than one type of information (e.g., patient self-reports combined with laboratory tests) and information from multiple sources (e.g., family members or friends) (Drake et al. 1993*a*). In addition, Rosenberg and colleagues (1996) recently developed a screening instrument, the Dartmouth Assessment of Lifestyle Instrument, that detects AOD-use disorders in psychiatric patients with greater accuracy than other instruments.

### Diagnosis

According to the DSM criteria, persistent alcohol use resulting in social, vocational, psychological, or physical problems should be considered abuse or dependence. This definition has several implications for diagnosing AOD-use disorders in severely mentally ill patients. For example, in psychiatric patients, who are more vulnerable to the effects of psychoactive drugs, use of relatively small amounts of AOD’s may result in psychological problems or relapse of the symptoms of mental illness or may evolve into an obvious use disorder (Dixon et al. 1990; Drake et al. 1989). Moreover, clinicians must be aware that in many patients with apparent dual diagnoses, AOD use may have induced the second psychiatric disorder (Lehman et al. 1994).

### Treatment Planning

During treatment planning, the clinician, together with the patient, reviews all data and specifies a strategy for further exploration or change of AOD-use behavior. Treatment planning includes a thorough biopsychosocial evaluation encompassing the following areas (Donovan 1988):

- Historical information and family history
- Current frequency and patterns of use
- Physiological factors
- Cognitive-behavioral expectancies related to the use of different drugs
- Environmental cues, social networks, and other social and behavioral patterns that sustain abuse
- Interrelationships between AOD use, medications, and psychiatric illnesses
- Previous attempts to control or treat AOD use.

Thus, treatment planning is a continuous, dynamic, and long-term process based on the clinician’s and patient’s collaboration.

## Treatment

For historical reasons, the mental health and AOD-abuse treatment systems in the United States are quite separate. Despite attempts to link the two treatment systems in traditional approaches to the care of patients with dual diagnoses, poor coordination between the systems may act as a treatment barrier for these patients (Osher and Drake 1996; Ridgely et al. 1987).

Over the past 15 years, however, mental health programs serving people with severe mental illnesses have moved toward integrating AOD-abuse treatment into a comprehensive treatment approach in which the same clinicians or teams of clinicians combine both mental health and AOD-abuse philosophies and treatment components (Carey 1996; Drake et al. 1993*b*; Drake and Mueser 1996; Lehman and Dixon 1995; Minkoff and Drake 1991). In addition to integrating mental health and AOD-abuse treatments, many of these programs also incorporate intensive case management approaches and outreach to facilitate engagement in treatment; comprehensive services and a team orientation; various types of group interventions; and a longitudinal, stagewise approach (Mueser and Noordsy 1996). A longitudinal, stage-wise approach is based on the findings that the recovery process typically occurs over years rather than weeks and often proceeds in several steps (e.g., the clients require motivational interventions before they are ready to participate in abstinence-oriented interventions).

Osher and Kofoed (1989) conceptualized four overlapping stages of AOD treatment for patients with severe mental illnesses: engagement, persuasion, active treatment, and relapse prevention. Engagement includes developing a trusting relationship, or working alliance, with the patient, whereas persuasion entails helping the patient to perceive and acknowledge the adverse consequences of AOD use in his or her life and develop motivation for recovery. During active treatment, the clinician helps the patient achieve stable recovery in the form of either controlled use or, preferably, abstinence. Relapse prevention focuses on helping the patient maintain stable recovery. During each stage, a range of treatment options are available, and the specific treatment plan should reflect the patient’s preferences. For example, some patients may benefit from participating in self-help programs (e.g., Alcoholics Anonymous) during active treatment or relapse prevention, whereas other patients may not. Clinicians employing a stagewise treatment approach may find it useful to consult a growing number of clinical guides describing various strategies for integrating mental health and AOD treatments for patients with dual diagnoses (e.g., Daley and Thase 1994; Evans and Sullivan 1990; Gold and Slaby 1991; Miller 1994; as well as the articles cited in the preceding paragraph).

Most programs integrating mental health and AOD treatment provide services on a long-term, outpatient basis in the community and attempt to minimize the time spent in inpatient, detoxification, or residential settings. Community-based treatment is emphasized because skills acquired by severely mentally ill patients in one setting (e.g., in a clinic) often fail to generalize to other settings (e.g., everyday life in the community). Thus, a premium is placed on working with patients in their natural environments. Nevertheless, brief treatment components in inpatient and detoxification settings can provide valuable opportunities for clinicians to establish or reestablish therapeutic relationships with patients during the engagement stage and to motivate patients to examine their AOD use and its possible consequences during the persuasion stage. Inpatient and outpatient services must be coordinated, however, in order to maximize long-term treatment gains.

Several recent studies indicate that integrated treatment programs combining AOD-abuse and mental health interventions within the same setting result in more positive outcomes than traditional, nonintegrated treatment systems (Drake et al. 1996*a*; Godley et al. 1994; Mueser et al. 1996). These studies show a steady reduction in AOD use, with the number of stably abstinent patients increasing with each year of consistent treatment. Other findings support the concept of treatment stages in the recovery process (McHugo et al. 1995). For example, in a recent study in New Hampshire, clients moved steadily through the stages of engagement, persuasion, active treatment, and relapse prevention, and approximately 50 percent of them achieved abstinence after 3 years of treatment (Mueser et al. 1996).

Not all investigators, however, have reported positive results of integrated treatment for dual-diagnosis patients. For example, Lehman and colleagues (1993) failed to find a beneficial effect of integrated treatment, possibly because the AOD-abuse measure they employed (i.e., the Addiction Severity Index) was not sufficiently sensitive to changes in AOD use in the severely mentally ill population studied (Corse et al. 1995). Also, not all integrated treatment approaches may be equally effective. Jerrell and Ridgely (1995) reported that an integrated treatment program with a focus on behavioral skills training reduced AOD abuse more effectively than a more traditional 12-step approach or a case management approach. The accumulated evidence suggests that providing integrated mental health and AOD treatment to dually diagnosed patients improves outcome compared with traditional, nonintegrated approaches. More research is needed, however, before definitive conclusions about the effectiveness of integrated treatment can be reached.

## Summary

Approximately 50 percent of clients with severe mental illnesses, such as schizophrenia and bipolar disorder, who are in community mental health settings develop AOD-use disorders during their lifetime. The rate probably is even greater among high-risk groups, such as young men with histories of violence or homelessness, and among patients in acute-care settings. AOD-use disorders among severely mentally ill patients are correlated with poor concurrent adjustment in several domains and with adverse short-term outcomes, including high rates of homelessness, hospitalization, and incarceration.

Clinicians often overlook AOD abuse among psychiatric patients. The use of standard screening and evaluation procedures could, however, greatly improve detection and diagnosis of AOD-related problems as well as treatment planning for this patient population. AOD-abuse treatment should be provided in stages over the long term by dual-diagnosis experts. Current research suggests that for patients with dual diagnoses, treatment approaches that integrate mental health and AOD treatment are particularly effective.

## Footnotes

1 The term “alcohol-use disorder” used in this article encompasses alcohol abuse and dependence as defined in the American Psychiatric Association’s *Diagnostic and Statistical Manual of Mental Disorders, Fourth Edition* (DSM–IV). The terms “alcohol-use disorder” and “alcohol abuse” are used interchangeably in this article. The definitions for these terms vary among the studies reviewed and frequently are based on earlier editions of the DSM. Definitions of other terms used in this article can be found in the glossary, p. 86.
